# *Haemophilus influenzae* serotype b seroprevalence in central Lao PDR before and after vaccine introduction

**DOI:** 10.1371/journal.pone.0274558

**Published:** 2022-09-15

**Authors:** Lisa Hefele, Jana Lai, Keoudomphone Vilivong, Toukta Bounkhoun, Valin Chanthaluanglath, Anisone Chanthongthip, Anne Balloch, Antony P. Black, Judith M. Hübschen, Fiona M. Russell, Claude P. Muller

**Affiliations:** 1 Department of Infection and Immunity, Luxembourg Institute of Health, Esch-sur-Alzette, Grand-Duchy of Luxembourg; 2 New Vaccines, Murdoch Children’s Research Institute, Parkville, Victoria, Australia; 3 National Centre for Epidemiology and Population Health, Acton, ACT, Australia; 4 Lao-Oxford Mahosot Hospital Wellcome Trust Research Unit, Vientiane Capital, Lao PDR; 5 LaoLuxLab, Institut Pasteur du Laos, Vientiane, Lao PDR; 6 Department of Paediatrics, The University of Melbourne, Victoria, Australia; 7 Asia-Pacific Health Research, Murdoch Children’s Research Institute, Melbourne, Victoria, Australia; Public Health England, UNITED KINGDOM

## Abstract

**Introduction:**

Vaccination has dramatically reduced invasive *Haemophilus influenzae* type b (Hib) disease worldwide. Hib vaccination was introduced in the Lao PDR in 2009, as part of the pentavalent vaccine. To contribute to the understanding of the epidemiology of Hib in Lao PDR and the protection levels before and after the introduction of the vaccination, we tested serum samples from existing cohorts of vaccine age-eligible children and unvaccinated adolescents for antibodies against Hib.

**Methods:**

Serum samples from 296 adolescents born before vaccine introduction and from 1017 children under 5 years (vaccinated and unvaccinated) were tested for anti-Hib antibodies by ELISA. Bivariate analyses were performed to investigate factors associated with long-term protection.

**Results:**

The vast majority of all participants showed evidence of short- (42.7%) or long-term (56.1%) protection against Hib. Almost all of the unvaccinated adolescents had antibody titers indicating short-term protection and almost half (45.6%) were long-term protected. Nearly all children (>99.0%) were at least short-term protected, even those that were unvaccinated or whose vaccination status was unknown. Among vaccinated children, participants vaccinated more than 1 or 2 years ago and with a mid-upper arm circumference z-score < -2 were less likely to be long-term protected.

**Discussion:**

Nearly all adolescents born before the introduction of Hib vaccination in the Lao PDR had antibody titers corresponding to at least short-term protection, indicating a high burden of Hib disease at that time. After vaccine introduction, all but four children (>99%) showed at least short-term protection. Possible explanations for the proportion of protected, yet apparently unvaccinated children, may be past infections, cross-reacting antibodies or faulty vaccination documentation. Our results highlight the need for robust surveillance and reporting of invasive Hib disease to determine the burden of disease despite vaccination.

## Introduction

*Haemophilus influenzae* type b (Hib) causes pneumonia and meningitis almost exclusively in children under 5 years of age [1]. Before widespread vaccination in 2000, Hib was responsible for at least 8.13 million cases of serious disease in children (<5 years) and 371 000 deaths globally. Vaccination has dramatically reduced invasive Hib disease worldwide [2]. The pentavalent vaccine DTPw-HepB-Hib (Diphtheria-Tetanus-Pertussis (whole cell)-Hepatitis B-*Haemophilus influenzae* type b), containing purified capsular Hib polysaccharide (PRP) conjugated to the tetanus toxoid (carrier protein), was introduced in Lao PDR in 2009, replacing the DTPw-HepB vaccine. DTPw-HepB-Hib is scheduled at 6, 10 and 14 weeks of age [3]. In 2018, the coverage with the DTPw-HepB-Hib vaccine was 84% in Lao PDR [4]. Immunity against Hib can be determined by measuring antibody levels against the PRP immunogenic component of Hib conjugate vaccines [5].

There are little data on the level of immunity against Hib or the burden of disease in the general population in Lao PDR. Two serosurveys conducted in Bolikhamxay province found that 66.4% and 71.7% of children 8 to 28 months old had long-term Hib antibody protection (>1μg/ml) in 2013/14 and 2017 respectively [6].

To contribute to the understanding of the epidemiology of Hib in Lao PDR and the protection levels before and after the introduction of the vaccination, we tested serum samples from existing cohorts of vaccine age-eligible children and unvaccinated adolescents in the Lao PDR for antibodies against PRP. We also investigated possible predictors for long-term protection.

## Methods

### Participants

#### Cohort 1: Unvaccinated adolescents

Serum samples of 296 students from Bolikhamxay province and Vientiane Capital, collected in 2018 in the framework of another study [7] were selected from a total of 779 students. All participants in this study were born before 2008, before Hib vaccine was introduced into the national immunization program and therefore were most likely not vaccinated against Hib. However, we cannot exclude the possibility that parents paid for the vaccination outside of the Lao PDR as this vaccine has been available on the private market in Thailand for many years. Students between the age of 11 and 18 years were selectively randomized for the same age and sex ratios in both provinces. Socio-economic data (i.e. district, age, sex, ethnicity, place of birth and number of household members) were collected using a standardized questionnaire. The study was approved by the Lao National Ethics Committee (Reference number 022/NECHR) and the Institutional Review Board of the Institut Pasteur du Laos (Reference number 9). All parents/guardians signed an informed consent form.

#### Cohort 2: Fully vaccinated children

This cohort consisted of 761 children from Vientiane (n = 178), Bolikhamxay (n = 228) and Khammouane (n = 355) province aged 9 to 50 months, recruited in the context of a previous study in 2013/14 [8]. All children had records of three doses of the pentavalent vaccine, as confirmed by reviewing the vaccination log books at the health care facilities. A subset (n = 140) of the anti-Hib data from Bolikhamxay was reported in a previous study [6]; the anti-Hib data from the entire cohort from Bolikhamxay (n = 228) were not reported before nor in conjunction with the other cohorts presented here. Socio-economic and health related data (i.e. district, age, sex, place of birth and date of vaccination etc.) were collected using a standardized questionnaire. In addition, the nutritional status of the children was determined by mid-upper arm circumference, weight for height, height for age, weight for age and body mass index z scores as described in the previous publication [8]. The study was approved by the Lao National Ethics Committee (NECHR2013-860). Informed consent was obtained from all parents of the children.

#### Cohort 3: Vaccine age-eligible acute respiratory infection contacts

256 children (<5 years of age) living in Vientiane Capital were purposively selected as contacts of children hospitalized with acute respiratory infection (ARI) between 2013 and 2016 [9]. A contact was defined as any child under the age of 5 coming into contact with the case in the preceding two weeks of hospitalization with ARI. The vaccination status of the children was assessed by reviewing the vaccination records in the parent-held mother child handbook (MCH) or via immunisation registers at the respective health centre. Participant information (i.e. age, sex, self-reported ethnicity, vaccination status, date of vaccination) were collected using a questionnaire. Ethics approval was received from the Royal Children’s Hospital (RCH) Human Research Ethics Committee (33177B; MCRI), Oxford Tropical Research Ethics Committee (1050–13; LOMWRU), WPRO Ethics Research Committee (2013.30.LAO.2.EPI), the Lao National Ethics Committee (2013–057) and the Human Research Ethics Committee (2016/770; ANU). Informed consent was obtained from all parents of the children.

### Laboratory analyses

#### Unvaccinated adolescents and vaccine age-eligible children (Cohort 1 and 2)

Serum samples were tested at the Institut Pasteur du Laos (IPL) for the presence of anti-PRP IgG using the commercial ELISA kit IMMUNOZYM (Progen) according to the manufacturer´s protocol. Antibody concentrations were derived from the optical density (OD) data using a standardized curve-fitting 4-parameter logistic method. Any sample above the calculation limit of the assay was given the value of 5.35 μg/ml, which corresponds to the concentration of the highest calibrator in this assay, for the purpose of analysis. Antibody titers below 0.15 μg/ml were considered as insufficient protection, titers between 0.15 and 1 μg/ml were considered as evidence for short-term protection and titers above 1 μg/ml were classified as sufficient immunity (long-term protection).

#### Vaccine age-eligible ARI contacts (Cohort 3)

These serum samples were tested for anti-Hib IgG at the Murdoch Children’s Research Institute, Victoria, Australia, following an established protocol [10, 11]. Microtiter plates were coated with *H*. *influenzae* type B oligosaccharide–human serum albumin conjugate (BEI Resources, Manassas, Virginia). Patient samples, the standard, anti-Hib capsular polysaccharide serum (lot 1983; FDA, Kensington, Maryland) and control anti-Hib human reference serum (National Institute for Biological Standards and Control, UK) were incubated on pre-coated plates. Horseradish peroxidase–conjugated anti-human immunoglobulin G (Millipore, Australia) and a tetramethylbenzidine (TMB) substrate solution (KPL, Gaithersburg, Maryland) were added for detection. The OD data were converted to antibody concentrations using KCjunior software (Bio-Tek Instruments Inc). Results were calculated using a standardized curve-fitting 4-parameter logistic method. The antibody titers were interpreted as described above.

### Data analyses

Data analyses were conducted using R statistical software [12] with the following packages: tidyverse [13], MASS [14], car [15], haven [16], lubridate [17], stringr [18] and epitools [19]. In bivariate analyses, odds ratio, 95% confidence intervals (CI) and p values were calculated to investigate factors associated with long-term protection. Chi-squared and Fisher´s exact tests were used as appropriate. Shapiro-Wilks goodness-of-fit test was used to assess the normality of data and the correlation between two numerical variables was assessed by calculating the Spearman rank correlation coefficient rho. A p value <0.05 was considered statistically significant.

## Results

### Participants’ characteristics

**Fig 1:**
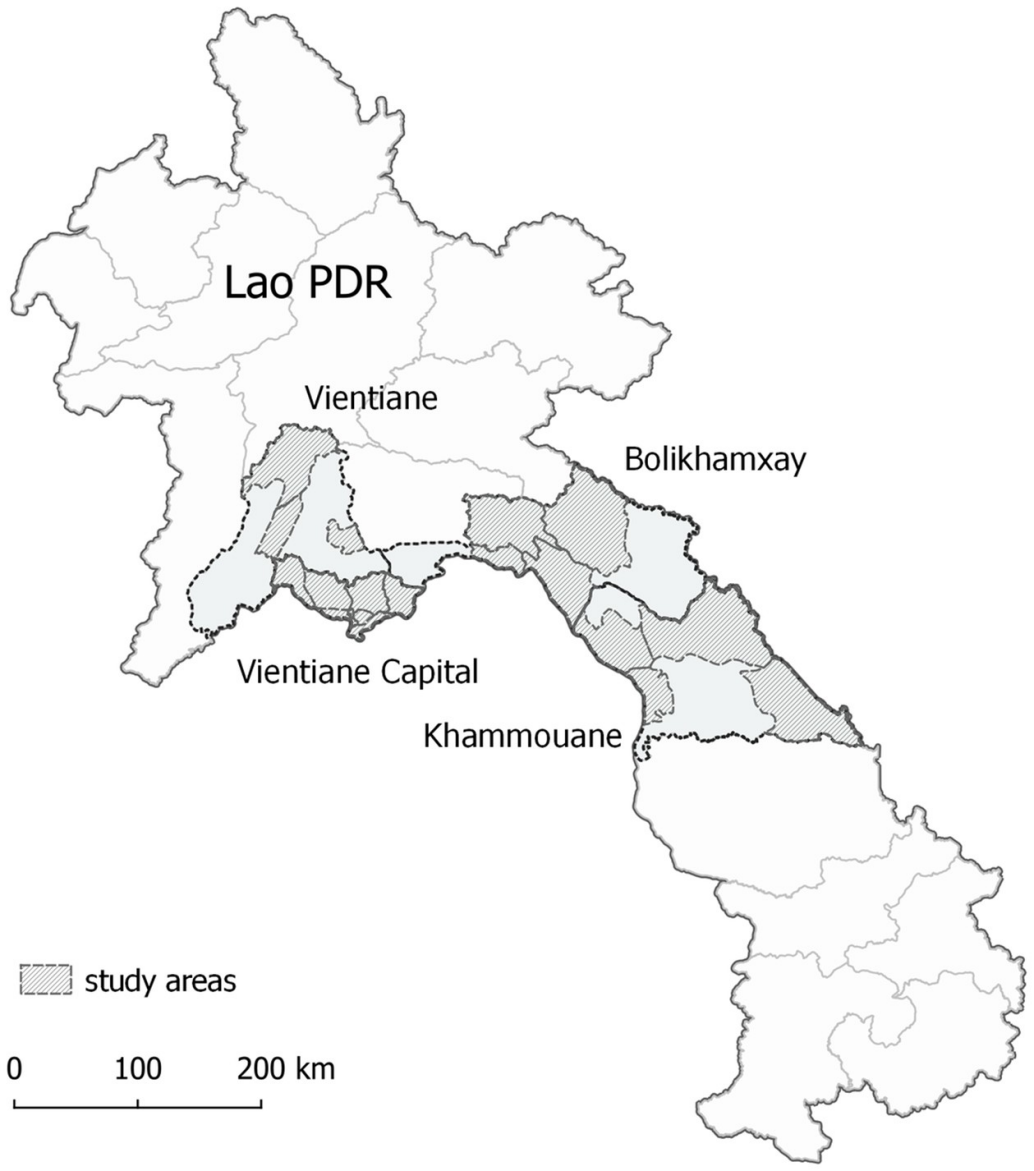
Map of study sites in Lao PDR. Districts in which samples were collected are highlighted. The map was created with QGIS (QGIS Development Team, 2018). The data regarding the administrative boundaries of Lao PDR was obtained from the Humanitarian Data Exchange website (https://data.humdata.org/dataset/lao-admin-boundaries, dataset provided by the National Geographic Department of Lao PDR, 2019). Projection used: EPSG 4326 –WGS 84.

In total, 1313 participants from the three cohorts were included (Table 1). The majority of the participants in cohort 1 and 3 were of Tai-Kadai ethnicity, which is the main ethnic group in Lao PDR. Information on ethnicity was not available for cohort 2. Serum samples of all three cohorts were collected in provinces located in central Lao PDR (Fig 1). Since all adolescents (cohort 1) were born before 2008, it was assumed they would not have received routine Hib vaccination. All children included in cohort 2 and half (52.3%) of the ARI contacts (cohort 3) had written records of a full course of the DTPw-HepB-Hib vaccine.

**Table 1:** Characteristics of study participants by cohort.

		Unvaccinated Adolescents (Cohort 1)	Vaccinated children (Cohort 2)	ARI contacts (Cohort 3)
Number of participants		296	761	256
Sample collection		2018	2013–2014	2013–2016
Age (years)	Median (IQR)	15 (13–17)	1.8 (1.2–2.3)	2.6 (2–3.5)
	Mean	14.6	1.9	2.7
Study Location n(%)	Vientiane Capital	148 (50.0)	0 (0)	256 (100)
	Vientiane Province	0 (0)	178 (23.4)	0 (0)
	Bolikhamxay Province	148 (50.0)	228 (30.0)	0 (0)
	Khammouane Province	0 (0)	355 (46.7)	0 (0)
Sex n(%)	Male	141 (47.6)	381 (50.1)	134 (52.3)
	Female	155 (52.4)	380 (49.9)	122 (47.7)
Ethnicity n(%)	Tai Kadai	283 (95.6)	NA	201 (78.5)
	Hmong-Mien & Mon-Khmer	13 (4.4)	NA	55 (21.5)
Hib vaccination status n(%)	Full course (documented)	NA	761 (100)	134 (52.3)
	Incomplete course	NA	0 (0)	11 (4.3)
	No vaccination	NA	0 (0)	24 (9.4)
	Unknown (no documentation)	296 (100)	0 (0)	87 (34.0)

Acute Respiratory Infection (ARI); IQR = Interquartile range; NA = not available.

### Prevalence of anti-Hib IgG

The vast majority of the participants in cohorts 1, 2 and 3 showed evidence of short- (42.7%) and long-term (56.1%) protection against Hib (S1 Table). Almost all (95.9%) of the unvaccinated adolescents (cohort 1) had an anti-Hib IgG titer >0.15μg/ml indicating at least short-term protection against Hib and almost half (45.6%) had anti-Hib IgG titers >1.0 μg/ml corresponding to long-term protection.

58.9% of the vaccinated children in cohort 2, all born after the introduction of the pentavalent vaccine, showed long-term protection (S1 Table). Long-term protection varied from 54.9% to 62.9% between the provinces and from 56.9% to 80% between the age groups. In ARI contacts (cohort 3), long-term protection rates ranged between 25.0% for unvaccinated to 67.2% for fully vaccinated children. None of the participants in this cohort had anti-Hib IgG titers below 0.15 μg/ml.

### Factors associated with long-term protection

Since nearly every participant in our study showed at least short-term protection, we could not assess associations between variables and short-term protection in either of the cohorts and instead focused on long-term protection as outcome.

**Fig 2:**
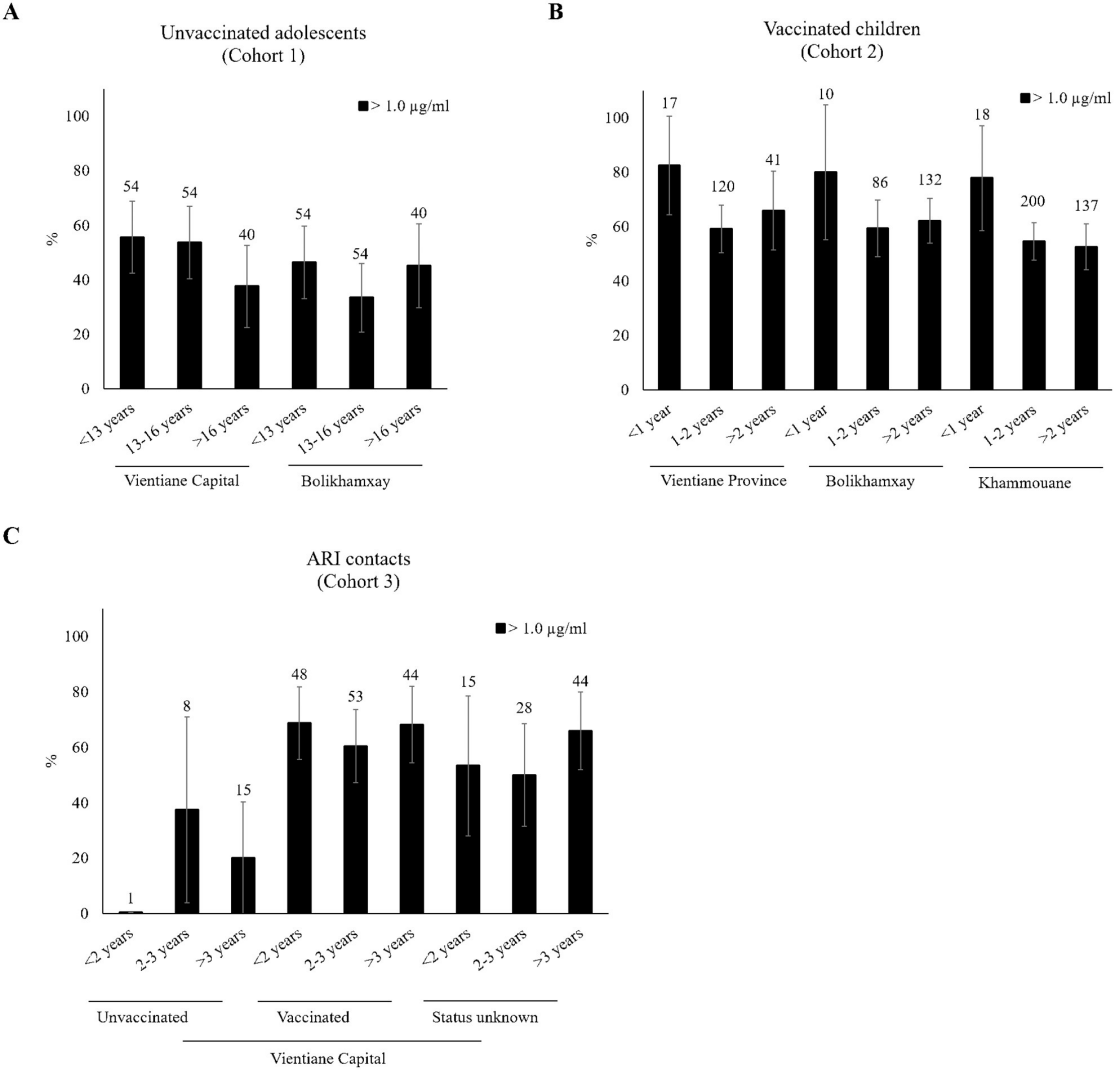
Proportion of participants with long-term protection (anti-Hib IgG >1 μg/ml) according to age group, vaccination status and study location. A) Unvaccinated adolescents (cohort 1). B) Vaccinated children (cohort 2). C) ARI contacts (cohort 3). Data is presented with 95% confidence intervals. The number above the bars represents the total number per group.

Despite some visible differences in the proportion of participants with long-term protection between age groups at the different locations, there was no statistical difference between the respective youngest and older age groups (Fig 2) in any of the cohorts. However, the proportion of long-term protection was higher in the cohort of children <5 years (cohort 2 and 3; n = 1017) compared to the adolescents (cohort 1; n = 296) (59.1% vs 45.6%; p<0.0001).

In the cohort of unvaccinated adolescents (cohort 1), being long-term protected (anti-Hib IgG >1 μg/ml) was not associated with study location (district or province), sex, age, place of birth, number of household members or ethnicity.

In vaccinated children (cohort 2), the odds of being long-term protected reduced over time. Participants vaccinated more than 1 or 2 years before the sample collection were less likely to have anti-Hib IgG >1.0 μg/ml (p = 0.01 and p = 0.02 respectively) (Table 2). Age and time since vaccination were also significantly correlated with antibody titers (rho = -0.08, p<0.05 and rho = -0.11, p<0.01, respectively). Furthermore, participants with a mid-upper arm circumference z-score < -2 were slightly less likely to be long-term protected (p = 0.03) (Table 2). None of the other factors were positively or negatively associated with long-term protection.

**Table 2:** Factors associated with long-term protection against Hib (>1μg/ml).

Cohort	Variable	Categories	Number of anti-Hib >1μg/ml / total number (%)	Bivariate analysis	
				OR [95% CI]	p-value
Cohort 2^a^	Time since vaccination	<1 year	156/236 (66.1)	1.0	
		1–2 years	210/378 (55.6)	0.64 [0.46–0.90]	0.01
		>2 years	71/133 (53.4)	0.59 [0.38–0.91]	0.02
		NA	14		
	Mid-upper arm circumference z-score	≥ -2	430/719 (59.8)	1.0	
		< -2	15/37 (40.5)	0.46 [0.23–0.90]	0.03
		NA	5		
Cohort 3^a^	Vaccination status	not vaccinated / unknown	57/111 (51.4)	1.0	
		vaccinated	97/145 (66.9)	1.91 [1.15–3.18]	0.01

^a^no other associations were found.

Hib = *Haemophilus influenzae* type B; OR = odds ratio; CI = confidence interval; NA = not available (missing data).

ARI contacts (cohort 3) who had been vaccinated (documented or undocumented) with DTPw-HepB-Hib were more likely to be long-term protected than participants with unknown vaccination status or who were not vaccinated (p = 0.01) (Table 2). Neither age nor sex, time since vaccination or ethnicity were associated with long-term protection.

## Discussion

In this study, we found high long-term protection rates in adolescents born before the introduction of the vaccine. Almost all (95.9%) unvaccinated adolescents in cohort 1 showed evidence of natural exposure to *H*. *influenzae* type b, corresponding as expected to the high burden of Hib circulation in an unvaccinated population. There are no data at a population level documenting the Hib carriage or disease in Lao PDR. Our findings are similar to other serosurveys showing high seropositivity rates before the introduction of the vaccination in 2009. For example in Kathmandu, Nepal, 20% of under-fives had anti-Hib levels >0.15μg/ml, which increased to 83% among 15–54 year olds [20].

Even after the introduction of Hib vaccination, all of the unvaccinated children in cohort 3 (ARI contacts) were at least short-term protected and 25% showed evidence of long-term protection (Table 2). The reason for this is unknown but suggests on-going exposure/infections, incomplete documentation of vaccination status, or cross-reactive antibodies with Hib. Being recruited as ARI contacts, these children may be more likely to have been exposed to Hib. Nevertheless, the results from our unvaccinated children (cohort 1 and 3) suggest that Hib is still present in the community.

After the introduction of Hib vaccination, all children in cohort 3 and virtually all (99.5%) children in cohort 2 showed at least short-term protection. The proportion of unprotected children in cohort 2 (vaccinated children) was less than 1%. All children in cohort 2 and 52.3% of the children in cohort 3 received all three doses of DTPw-HepB-Hib vaccine; however, 41.1% in cohort 2 and 35.2% of the vaccinated children in cohort 3 had antibody titers insufficient for long-term protection. We reported low levels of antibodies against diphtheria, tetanus and hepatitis B, (components of the same pentavalent vaccine) without a clear explanation in cohort 2 previously [8]. A follow-up study in 2017 revealed that vaccine immunogenicity for diphtheria, tetanus and hepatitis B increased by around 20% compared to the study in 2013/14 [6]. However, the increase by 5% of long-term protection in response to the Hib component in 2017 was minimal (from 66.4% to 71.7%). Another reason for the large proportion of short-term protected children could be rapid waning of anti-Hib antibodies. Indeed, time since vaccination was associated with having a lower antibody titer in cohort 2 in the present study. Hib antibody waning has been reported previously and the introduction of a booster dose into the vaccination schedule has been discussed [2, 21, 22]. Furthermore, since data quality in the health sector has been of concern in Lao PDR before, we cannot exclude the possibility that the participants had not received the vaccination despite having documentation; or that they received the vaccination without having documentation [23].

None of the participant characteristics in cohort 1 was associated with long-term protection, indicating that at least in this setting, socio-economic factors did not seem to influence Hib infection. There were also no significant differences in long-term protection rates by location, ethnicity or most of the other factors within cohort 2 and 3, except for vaccination, time since vaccination and one of the nutritional parameters. The mid-upper arm circumference z-score < -2 was associated with shorter protection. Thus, malnutrition does not seem to have a major role in the response to Hib conjugate vaccine in this setting. A weak association between seroprotection against diphtheria and malnutrition was reported in cohort 2 before [8].

More data are needed to evaluate whether the vaccination led to a reduction in Hib infection in children. Other countries have reported a decrease of confirmed Hib related diseases after vaccine introduction using a primary three dose schedule as recommended by WHO [24–26]. A study in Kenya analysed 15 years of Hib surveillance data and found that the vaccine reduced the risk of Hib disease by 93% over this period. Additionally, they found that eight years after the introduction of the vaccine, 79% of children in the disease risk group, aged 4–35 months, had antibodies at levels indicating long-lasting protection [24]. In The Gambia, 13 years after the Hib vaccination was introduced as a primary 3-dose schedule, the vaccine remained highly effective in controlling invasive Hib disease [11]. Clinical data in addition to the monitoring of vaccine-induced protection rates could provide helpful information whether a booster is needed, using this study as baseline. Nevertheless, where Hib vaccine evaluations have been undertaken in low- and middle-income countries, a booster is not required to reduce Hib disease and therefore it is recommended that the Lao PDR schedule should not change at this time [24].

Limitations of our study may result from the different study designs of the original studies and laboratory methods. Participants in cohorts 2 and 3 were not selected randomly. Information regarding infection with *H*. *influenza* type b among the participants or other clinical information regarding the participants in the cohorts were not available. Furthermore, we assumed that participants born before vaccine introduction would be unvaccinated and there is no information regarding vaccination status of 34% of participants in cohort 3. This uncertainty may have affected the interpretation of our results. Lastly, our findings are from Central areas of the Lao PDR. Hib prevalence may vary between regions of Lao PDR and/or between different ethnic groups.

## Conclusion

Our findings indicate that the circulation of Hib was high in Lao PDR before the introduction of the vaccine and continues to be high in unvaccinated children. Indeed, after vaccine introduction, all vaccinated children, but also all others showed serological markers of vaccination/past infection and protection. Thus, robust surveillance and systematic reporting of invasive Hib cases is required to determine the current burden of disease despite vaccination.

## Supporting information

S1 Table Anti-Hib IgG prevalence according to participants’ characteristics for each study cohort. (DOCX)
