# Supplementary material for: Haemophilus influenzae serotype b seroprevalence in central Lao PDR before and after vaccine introduction
Source: PLoS One. 2022 Sep 15;17(9):e0274558. doi: 10.1371/journal.pone.0274558 (PMC9477263; doi:10.1371/journal.pone.0274558)
Supplement: S1 Table — (DOCX) [file pone.0274558.s001.docx]

**S1 Table Anti-Hib IgG prevalence according to participants’ characteristics for each study cohort**

| **Predictor** | **Categories** | **n (% of total N)** | **Hib serology** | | |
| --- | --- | --- | --- | --- | --- |
|  |  |  |  |  |  |
|  |  |  | **<0.15 µg/ml** | **0.15-1.0 µg/ml** | **>1.0 µg/ml** |
| **All participants** | | 1313 (100) | 1.2 | 42.7 | 56.1 |
| **Unvaccinated adolescents**  **(Cohort 1; N=296)** | |  |  |  |  |
| All |  | 296 (100) | 4.1 | 50.3 | 45.6 |
| Province | Vientiane Capital | 148 (50.0) | 2.0 | 48.0 | 50.0 |
|  | Bolikhamxay | 148 (50.0) | 6.1 | 52.7 | 41.2 |
| District | Vientiane Capital | 148 (50.0) | 2.0 | 48.0 | 50.0 |
|  | Paksan | 81 (27.4) | 9.9 | 50.6 | 39.5 |
|  | Pakkading | 67 (22.6) | 1.5 | 55.2 | 43.3 |
| Sex | Male | 141 (47.6) | 2.1 | 48.9 | 48.9 |
|  | Female | 155 (52.4) | 5.8 | 51.6 | 42.6 |
| Age | <13 years | 108 (36.5) | 6.5 | 42.6 | 50.9 |
|  | 13-16 years | 108 (36.5) | 1.9 | 54.6 | 43.5 |
|  | >16 years | 80 (27.0) | 3.8 | 55.0 | 41.3 |
| Ethnicity | Tai Kadai | 283 (95.6) | 4.2 | 50.9 | 44.9 |
|  | Hmong-Mien & Mon-Khmer | 13 (4.4) | 0.0 | 38.5 | 61.5 |
| Place of birth | Central Hospital in Vientiane | 71 (24.0) | 1.4 | 45.1 | 53.5 |
|  | Other & Unknown | 225 (76.0) | 4.9 | 52.0 | 43.1 |
| N of household members | ≤4 | 88 (29.7) | 8.0 | 50.0 | 42.0 |
|  | >4 | 208 (70.3) | 2.4 | 50.5 | 47.1 |
| **Vaccinated children**  **(Cohort 2; N=761)** | |  |  |  |  |
| All |  | 761 (100) | 0.5 | 40.6 | 58.9 |
| Province | Vientiane Province | 178 (23.4) | 0.0 | 37.1 | 62.9 |
|  | Bolikhamxay | 228 (30.0) | 0.0 | 38.2 | 61.8 |
|  | Khammouane | 355 (46.6) | 1.1 | 43.9 | 54.9 |
| Age | <1 year | 45 (5.9) | 0.0 | 20.0 | 80.0 |
|  | 1-2 years | 406 (53.4) | 0.2 | 42.9 | 56.9 |
|  | >2 years | 310 (40.7) | 1.0 | 40.6 | 58.4 |
| Sex | Male | 381 (50.1) | 0.5 | 40.9 | 58.5 |
|  | Female | 380 (49.9) | 0.5 | 40.3 | 59.2 |
| Place of birth | Home | 234 (30.7) | 1.3 | 41.9 | 56.8 |
|  | Health Care Facility | 524 (68.9) | 0.2 | 39.9 | 59.9 |
|  | Unknown | 3 (0.4) | 0.0 | 66.7 | 33.3 |
| Time since vaccination | <1 year | 236 (31.0) | 0.0 | 33.9 | 66.1 |
|  | 1-2 years | 378 (49.7) | 0.5 | 43.9 | 55.6 |
|  | >2 years | 133 (17.5) | 1.5 | 45.1 | 53.4 |
|  | NA | 14 (1.8) | 0.0 | 21.4 | 78.6 |
| Weight for height z-score | ≥ -2 | 684 (89.9) | 0.6 | 40.1 | 59.4 |
|  | < -2 | 61 (8.0) | 0.0 | 50.8 | 49.2 |
|  | Unknown | 16 (2.1) | 0.0 | 25.0 | 75.0 |
| Height for age z-score | ≥ -2 | 430 (56.5) | 0.5 | 39.1 | 60.5 |
|  | < -2 | 306 (40.2) | 0.7 | 43.1 | 56.2 |
|  | Unknown | 25 (3.3) | 0.0 | 36.0 | 64.0 |
| Weight for age z-score | ≥ -2 | 590 (77.5) | 0.3 | 40.0 | 59.7 |
|  | < -2 | 166 (21.8) | 1.2 | 42.8 | 56.0 |
|  | Unknown | 5 (0.7) | 0.0 | 40.0 | 60.0 |
| Mid-upper arm circumference z-score | ≥ -2 | 719 (94.5) | 0.6 | 39.6 | 59.8 |
|  | < -2 | 37 (4.9) | 0.0 | 59.5 | 40.5 |
|  | Unknown | 5 (0.7) | 0.0 | 40.0 | 60.0 |
| Parasite infection | No | 383 (50.3) | 0.3 | 40.5 | 59.3 |
|  | Yes | 84 (11.0) | 1.2 | 45.2 | 53.6 |
|  | Unknown | 294 (38.6) | 0.7 | 39.5 | 59.9 |
| **ARI contacts**  **(Cohort 3; N=256)** | |  |  |  |  |
| All |  | 256 (100) | 0.0 | 39.8 | 60.2 |
| Age | >2 | 64 (25.0) | 0.0 | 35.9 | 64.1 |
|  | 2-3 years | 88 (34.4) | 0.0 | 42.0 | 58.0 |
|  | >3 years | 104 (40.6) | 0.0 | 40.4 | 59.6 |
| Sex | Male | 134 (52.3) | 0.0 | 37.3 | 62.7 |
|  | Female | 122 (47.7) | 0.0 | 42.6 | 57.4 |
| Ethnicity | Tai Kadai | 201 (78.5) | 0.0 | 40.8 | 59.2 |
|  | Hmong-Mien & Mon-Khmer | 55 (21.5) | 0.0 | 36.4 | 63.6 |
| Vaccination with DTPw-HepB-Hib | Full course (documented) | 134 (52.3) | 0.0 | 32.8 | 67.2 |
|  | Incomplete course | 11 (4.3) | 0.0 | 36.4 | 63.6 |
|  | No | 24 (9.4) | 0.0 | 75.0 | 25.0 |
|  | Unknown (no documentation) | 87 (34.0) | 0.0 | 41.4 | 58.6 |
| time since vaccination | ≤2 years | 71 (27.7) | 0.0 | 29.6 | 70.4 |
|  | >2 years | 64 (25.0) | 0.0 | 35.9 | 64.1 |
|  | NA | 121 (47.3) | 0.0 | 47.9 | 52.1 |
| N = number; NA = not available; Hib = Haemophilus influenza type B; DTPw-HepB-Hib Diphtheria-Tetanus-Pertussis-Hepatitis B-Haemophilus influenzae type B vaccine | | | | | |
